# Reversal of Ovarian Cancer Cell Lines Multidrug Resistance Phenotype by the Association of Apiole with Chemotherapies

**DOI:** 10.3390/ph13100327

**Published:** 2020-10-21

**Authors:** Carolina Afonso de Lima, Ian Lucas de Souza Bueno, Stanley Nunes Siqueira Vasconcelos, Juliana Mozer Sciani, Ana Lúcia Tasca Gois Ruiz, Mary Ann Foglio, João Ernesto de Carvalho, Giovanna Barbarini Longato

**Affiliations:** 1 Research Laboratory in Molecular Pharmacology and Bioactive Compounds, São Francisco University, Bragança Paulista, São Paulo 12916-900, Brazil; carolinaafonsolima@hotmail.com (C.A.d.L.); ianlucasbueno@gmail.com (I.L.d.S.B.); 2 Center for Medicinal Chemistry (CQMED), University of Campinas (UNICAMP), Campinas, São Paulo 13083-875, Brazil; stanleynsv@gmail.com; 3 Center for Molecular Biology and Genetic Engineering (CBMEG), University of Campinas (UNICAMP), Campinas, São Paulo 13083-875, Brazil; 4 Structural Genomics Consortium, Department of Genetics and Evolution, Institute of Biology (IB), University of Campinas (UNICAMP), Campinas, São Paulo 3083-866, Brazil; 5 Laboratory of Multidisciplinary Research, São Francisco University, Bragança Paulista, São Paulo 12916-900, Brazil; juliana.sciani@usf.edu.br; 6 LAFTEx Faculty of Pharmaceutical Science, University of Campinas, Campinas, São Paulo 13083-859, Brazil; ana.ruiz@fcf.unicamp.br (A.L.T.G.R.); maryann.foglio@fcf.unicamp.br (M.A.F.); carvalho@fcf.unicamp.br (J.E.d.C.)

**Keywords:** multidrug resistance, glycoprotein-P, apiole, in silico, druglikeness

## Abstract

Multidrug resistance (MDR) is the main obstacle in anticancer therapy. The use of drug combinations to circumvent tumor resistance is a well-established principle in the clinic. Among the therapeutic targets, glycoprotein-P (P-gp), an energy-dependent transmembrane efflux pump responsible for modulating MDR, is highlighted. Many pharmacological studies report the ability of calcium channel blockers to reverse tumor resistance to chemotherapy drugs. Isolated for the first time from parsley, the phenylpropanoid apiole is described as a potent calcium channel inhibitor. Taking this into account, herein, the ability of apiole to potentiate the action of well-established chemotherapeutics in the clinic, as well as the compound’s relationship with the reversal of the resistance phenomenon by blocking P-gp, is reported. The association of apiole with both chemotherapeutic drugs doxorubicin and vincristine resulted in synergistic effect, in a concentration-dependent manner, as evaluated by the concentration reduction index. Molecular docking analysis demonstrated the affinity between apiole and the active site of P-gp, corroborating the inhibitory effect. Moreover, apiole demonstrated druglikeness, according to ADME analysis. In conclusion, apiole possibly blocks the active P-gp site, with strong binding energy, which, in turn, inhibits doxorubicin and vincristine efflux, increasing the antiproliferative response of these chemotherapeutic agents.

## 1. Introduction

Including more than 100 different diseases, cancer is characterized by disordered cells growth with the ability to invade tissues and organs; some types of tumor cells can also spread to other body parts in a process called metastasis [1]. According to the International for Union Against Cancer (UICC), in 2020, an estimate of 15 million new cases of cancer will occur and this disease will be responsible for 12% of deaths worldwide, considering the non-infectious causes of death.

Multidrug resistance (MDR) is one of the main obstacles of anticancer treatment and believed to cause treatment failure in over 90% of patients with metastatic cancer [2]. Although most patients initially respond to chemotherapy, a significant percentage of them relapse, developing resistance to a broad spectrum of structurally unrelated drugs that do not share a common target [3].

One of the major causes of multidrug resistance is the enhanced efflux of drugs by membrane ABC (ATP-binding cassete) transporters. Targeting ABC transporters projects a promising approach to eliminating or suppressing drug resistance in cancer treatment [4]. Among these members, P-glycoprotein (P-gp, also referred to as multidrug resistance protein 1, MDR1, or ABCB1) is the best characterized efflux pump that mediates cancer MDR [5].

Structurally, P-gp is formed by four-domain architecture consisting of two cytoplasmic nucleotide-binding domains (NBDs) that bind and hydrolyse ATP and two transmembrane domains (TMDs) that recognize and transport substrates [6]. Abundant in gastrointestinal and hepatic systems, P-gp acts as a drug efflux pump capturing drug molecules in cytoplasma and returning them to the cell interstice [7]. Among general strategy to overcome multidrug resistance is the coadministration of P-gp chemical inhibitors with anticancer drugs [8].

Apiole is a phenylpropanoid found in the roots, seeds and leaves of parsley (Apiaceae), but also in Lauraceae and Piperaceae species [9]. This compound is popularly known as an abortifacient. Medicinal properties such as antioxidant, antibacterial, antihyperlipidemic, antihypercholesterolemic, antimycobacterial, chemopreventive, antidiabetic and anti-inflammatory have been described. Beneficial uses reported for apiole include the treatment of uterus diseases and cervical ectropio [10]. There are few studies in the scientific field showing that apiole can suppress colorectal cancer cell growth [11,12,13], in addition to being a potent calcium channel inhibitor [14,15].

Considering that calcium channel blockers have been shown to be substrates for P-gp and potent MDR reversers [16], this study aimed to evaluate the action of apiole in adjuvant treatment with well-established chemotherapy drugs used in the clinic, such as doxorubicin and vincristine, on ovarian tumor cell lines.

## 2. Results

### 2.1. Apiole Identification

**Figure 1:**
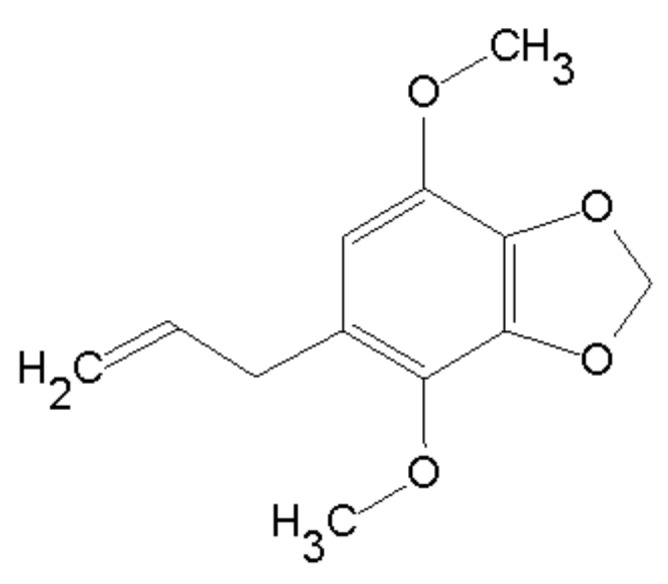
Apiole chemical structure.

The nuclear magnetic resonance (NMR) spectral data (Table 1) obtained for isolated compound was in accordance to previously reported data [17], confirming the structure (Figure 1). Furthermore, melting point ranged between 29.4 and 29.6, consistent with Pubchem [18]. The compound purity is above 98%, detected within the detection limit of equipments.

### 2.2. Antiproliferative Activity of Apiole, Doxorubicin and Vincristine

**Figure 2:**
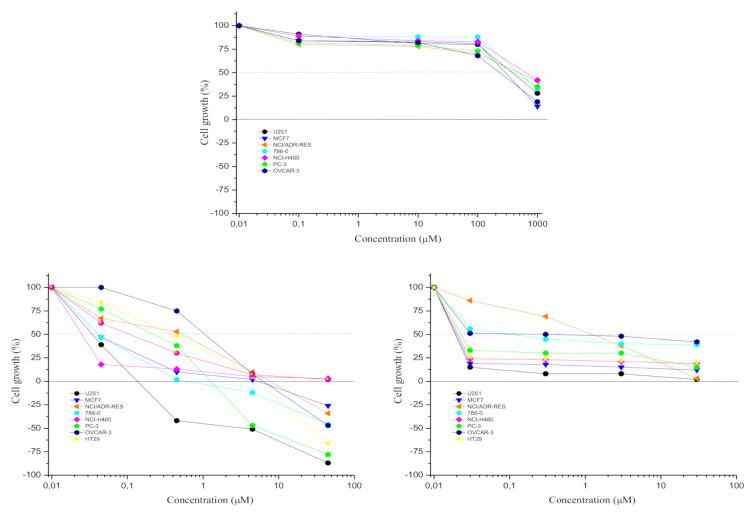
Antiproliferative profiles of apiole (**A**) and the chemotherapeutic drugs doxorubicin (**B**) and vincristine (**C**) against a human cell line panel after 48 h exposure. Human tumor cell lines: U251—central nervous system, glioblastoma; MCF7—breast, adenocarcinoma; NCI/ADR-RES—multidrug-resistant ovarian adenocarcinoma; 786-0—renal adenocarcinoma; NCI-H460—lung, large cell carcinoma; PC-3—prostate, adenocarcinoma; OVCAR-3—ovarian adenocarcinoma; HT29—colon, adenocarcinoma. Concentration range: apiole (1–1000 µM); doxorubicin (0.045–45 µM) and vincristine (0.03–30 µM).

The apiole by itself displayed low cytostatic activity (Figure 2A) with high GI_50_ values (Table 2). As expected, doxorubicin and vincristine inhibited cell proliferation (Figure 2B,C and Table 2). The different profiles between doxorubicin and vincristine are representative of the differences in the mechanism of action of these two drugs. Considering the antiproliferative effect (Table 2), the most resistant cell line for both doxorubicin and vincristine was NCI/ADR-RES (multidrug resistant ovarian adenocarcinoma, GI_50_ = 4.63 and 4.71 µM, respectively).

### 2.3. Association of Apiole with Doxorubicin on NCI/ADR-RES Cell Line

**Figure 3:**
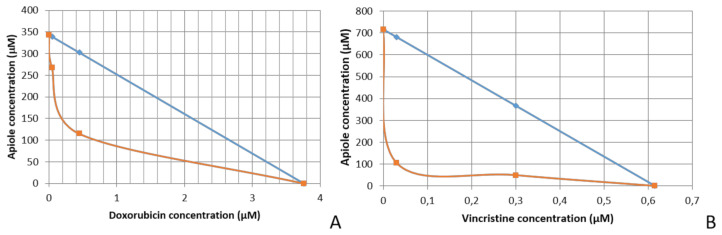
Isobolograms representing the synergistic interaction of the apiole with chemotherapeutic drugs doxorubicin (**A**) and vincristine (**B**) on NCI/ADR-RES cell line, after 48 h of incubation.

**Figure 4:**
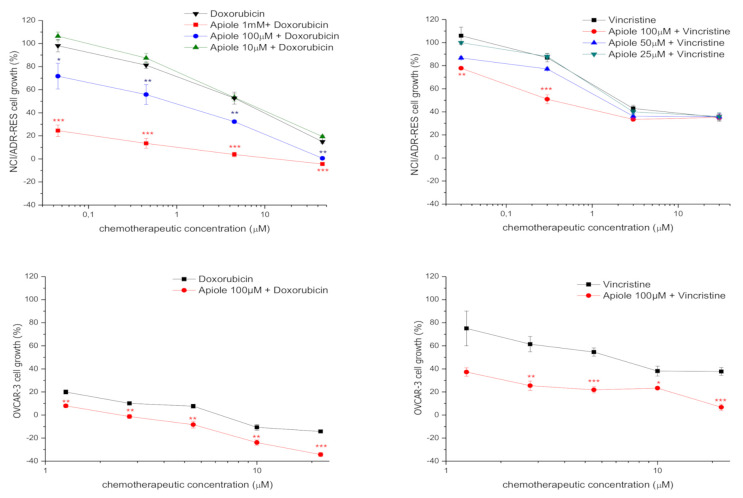
Influence of apiole on the antiproliferative profiles of the chemotherapeutic drugs doxorubicin and vincristine against human ovarian tumor cell line panel after 48 h exposure. (**A**) Human tumor cell line: multidrug-resistant ovarian adenocarcinoma cell line NCI/ADR-RES; Treatments: apiole (10, 100 and 1000 µM), doxorubicin (0.045–45 µM); (**B**) Human tumor cell line: multidrug-resistant ovarian adenocarcinoma cell line NCI/ADR-RES; Treatments: apiole (25, 50 and 100 µM), vincristine (0.03–30 µM); (**C**) Human tumor cell line: ovarian adenocarcinoma cell line OVCAR-3; Treatments: apiole (100 µM), doxorubicin (1.25 to 20 μM). (**D**) Human tumor cell line: ovarian adenocarcinoma cell line OVCAR-3; Treatments: apiole (100 µM), vincristine (1.25 to 20 μM). Statistical analysis by one-way ANOVA followed by Tukey’s test (* *p* < 0.05; ** *p* < 0.01; *** *p* < 0.001, related to chemotherapeutic drug alone). The experiments were carried out in triplicate.

The association of apiole and doxorubicin resulted in synergistic effect (Figure 3A). Concentration reduction index (CRI) was calculated by the GI_50_ doxorubicin/GI_50_ doxorubicin+apiole ratio. The synergistic effect was evidenced by CRI values higher than 1 (synergistic indicator). The best results for doxorubicin were obtained when apiole was associated at 1 mM (GI_50_ < 0.045 µM, CRI > 102.89) and at 100 µM (GI_50_ = 0.55 µM, CRI = 8.42). At the lowest concentration (10 µM) apiole did not improve the cytostatic effect of doxorubicin (Table 3, Figure 4A).

### 2.4. Association of Apiole with Vincristine on NCI/ADR-RES Cell Line

The association of apiole and vincristine also resulted in synergistic effect (Figure 3B). Considering the GI_50_ and the CRI values, the best results for vincristine were obtained when apiole was associated at 100 µM (GI_50_ = 0.75 µM, CRI = 6.28) and at 50 µM (GI_50_ = 2.70 µM, CRI = 1.74). At the lowest concentration (25 µM) apiole did not improve the cytostatic effect of vincristine (Table 4, Figure 4B).

### 2.5. Association of Apiole-Doxorubicin with Apiole-Vincristine on OVCAR-3 Cell Line

Based on the results for NCI/ADR-RES cells, the association of apiole, at 100 µM, with doxorubicin and vincristine was evaluated against one ovarian adenocarcinoma cell line (OVCAR-3) that did not express multidrug resistance phenotype. As expected, apiole promoted a reduction on GI_50_ for both doxorubicin (GI_50_ = 0.32 µM, CRI = 2.2) and vincristine (GI_50_ = 0.43 µM, CRI = 2.8) association (Table 5, Figure 4C,D, respectively), however, this effect was less pronounced in OVCAR-3 cell line in comparison to NCI/ADR-RES.

### 2.6. Molecular Docking

**Figure 5:**
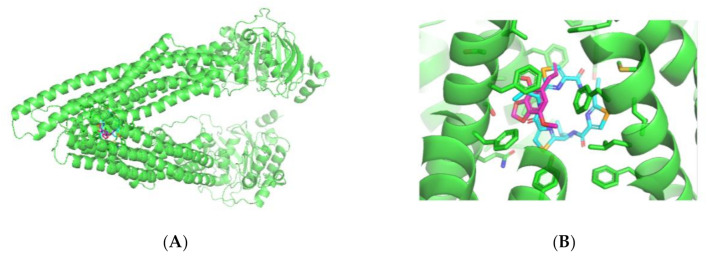
Graphical representation of binding mode of P-glycoprotein, apiole and macrocycle (**A**). Detail of the bindind pocket of ligands to the P-glycoprotein, showing its sites occupied by Apiole and macrocycle (**B**). Ligands are represented by the pink (apiole) and blue (macrocycle) color; P-gp is represented by the green color.

Molecular docking was performed using P-glycoprotein (P-gp) (Acession code 3G60 in the RCSB Protein Data Bank) and the apiole ligand. Apiole was placed in the center of the two membrane-embedded transmembrane domains (TMDs), where there is a large binding surface, with high binding energy—5.2 kcal.mol, being considered a stable force between apiole and the P-gp binding site (Figure 5A). Macrocycle, a compound already reported in the literature that has the potential to bind to one of the active sites of P-gp, was used as reference. Apiole bound at the same pocket site as the reference compound (Figure 5B), through the glycoprotein exit pathway, which indicates blockage of substract binding and transport [19]. Although both ligands do not share the same amino acid residue interaction (Phe332 for macrocycle and Phe 728/Tyr 303 for apiole), it is important to mention that several other ligands have specific amino acid binding to P-gp, with the same inhibitory effect [20,21].

### 2.7. In Silico PK

Pharmacokinetic parameters were calculated based on the apiole structure (Table 6) and compared to the criteria established by Lipinski to determine the druglikeness [22]. Data demonstrated that apiole follows all the Lipinski’s rules, except TPSA, although the value is close to the lower limit criteria. Nevertheless, no alert was considered for medicinal chemistry and 0 violation for druglikeness. Moreover, the molecule was classified as soluble in water (2.42e-01 mg/mL).

## 3. Discussion

The development of MDR is one of the main obstacles in chemotherapy treatment against tumors [23]. This resistance is multifactorial, and may result, among other factors, from the activation or overexpression of proteins from ABC transporter family (ATP Binding Cassette). Resistance to chemotherapeutic drugs promoted by P-gp, a well characterized ABC-transporter, is the most studied form of resistance in vitro, in vivo and in the clinic and, consequently, a large number of compounds are investigated for their abilities to affect the phenomenon [24].

Many drugs, including calcium channel blockers, prevent P-gp phosphorylation, which is necessary for the protein’s pumping function. By blocking this resistance mechanism, the chemotherapeutic agent is able to produce antiproliferative effect repeatedly [25]. As a calcium channel blocker [11], initially it was hypothesized that apiole’s mechanism of action was the same as first, second and third-generation modulators, binding to the P-gp present in the ovarian tumor cells membrane, inhibiting the extrusion mechanism of chemotherapeutic agents, and consequently, potentiating the substance’s antiproliferative activity. Molecular docking analysis gave insights that apiole has an affinity to the active site of the P-glycoprotein, evidenced by the efficient binding force to this protein. Such results suggest that this compound may inhibit P-gp.

The potential use of calcium channel blocking drugs in cancer therapy to revert MDR [26] and the affinity to P-gp observed for apiole by molecular docking prompted the antiproliferative study associating apiole and two chemotherapeutic drugs frequently used in the clinic (doxorubicin and vincristine) in two ovarian cancer cell lines (NCI/ADR-RES, which expresses the phenotype of resistance to multiple drugs, characterized by the high expression of P-gp [27]; and OVCAR-3).

The results herein presented revealed a synergistic effect when apiole was associated with doxorubicin and vincristine, in a concentration-dependent manner, in both ovarian tumor lines, being more effective in the resistant cell line NCI/ADR-RES, which reinforced the involvement of apiole with P-gp.

Considering the potential use clinical use of apiol in association with chemotherapeutic drugs, we evaluated in silico some parameters for druglikeness according Lipinski’s criteria. These evaluations demonstrated that apiole presented high water solubility suggesting compatibility with biologic fluids associated with adequated balance between and the hydrogen-bond acceptors and donors are adequate for water and target interactions [28]. The calculated logP for apiol (2.85) suggested a good ability to cross plasmatic membranes which might reflect its high gastrointestinal absorption. This parameter reflects the possibility of one substance to reach the molecular target at effective concentration after oral administration [29]. Besides, the in silico evaluation has shown that apiole has no ability to inhibit the main hepatic enzymes (P450 complex) suggesting a lower risk of drug interaction mediated by interference drug metabolism.

The low toxicity predicted for apiole was confirmed by previous studies. In mice tumor models [12,13], apiole promoted antitumor effects without clinical signs of toxicity up to 30 mg/kg, three times per week, by intraperitoneal route. In comparison to other allylbenzenes derivatives, apiole was found as the less potent genotoxic agent [30,31]. A careful toxicological study should be considered for apiole, once this molecule is popularly known by its abortive and toxic effects.

Despite considerable in vitro success, there are no compounds currently available to block P-gp–mediated resistance in the clinic. The failure may be attributed to toxicity, adverse drug interaction, and numerous pharmacokinetic issues [32].

The most important first generation calcium channel blocker, verapamil, failed in clinical trials because the plasmatic concentration required to reverse MDR (6–10 µM) was higher than that required to promote effects on the cardiovascular system (1 to 2 µM) [33]. These significant cardiovascular adverse effects still limit in the clinic use of verapamil [34]. Despite showing better pharmacological ant toxic profiles, the second-generation of P-gp modulators, represented by valspodar and biricodar, also failed in clinical trails mainly because they are substrates for cytochrome P450 superfamily, especialy CYP3A4. Considering the key role of CYP3A4 in the metabolism and excretion of cytotoxic agents, the coadministration of a potential modulator of both P-gp and CYP3A4 might promote unwanted changes on pharmacokinetic parameters of the chemotherapeutic agent leading to toxic drug-drug interactions. A third generation of P-gp inhibitors is represented by tariquidar, which has high affinity to P-gp at nanomolar concentrations. This generation of P-gp inhibitors has been examined in preclinical and clinical studies; however, the trials have largely failed to demonstrate an improvement in therapeutic efficacy [19,32,33,34]. Therefore, the search for resistance reversal modulators that do not present adverse effects to be introduced in the clinic has been incessant. New strategies to find forth generation of P-gp inhibitors have been used by investigators, such as the “return” to natural products [19].

In this context, our in silico and in vitro results together with the in vitro and in vivo data available in literature pointed out that apiole could be a promisor alternative to reverse MDR in cancer chemotherapy with low potential to elicit unwanted drug-drug interactions. Further non-clinical studies should be done to complement both pharmacological (adjuvant calcium channel blocker in cancer therapy) and toxicological evaluation of apiole. To the best of our knowledge, this is the first article to report the chemoreversion property of apiole.

## 4. Material and Methods

### 4.1. Isolation of Apiole Obtained from Piper Regnellii (Miq.) C. DC. var. Regnellii Leaves

Apiole was isolated from Piper regnellii (Miq.) C. DC. var. regnellii leaves. Briefly, Piper regnellii (Miq.) C. DC. var. regnellii leaves (voucher number CPMA 221) were collected at Centro Pluridisciplinar de Pesquisas Químicas, Biológicas e Agrícolas (CPQBA, Universidade Estadual de Campinas, São Paulo, Brazil). From the dry milled leaves (218 g) the dichloromethane crude extract was obtained (DCE, 6% yield) and fractionated by column chromatography using silica gel with an increasing gradient of hexane, dichloromethane, and methanol, providing 48 fractions (50 mL each one). The resulting fractions were grouped (FRA-FRG) according to thin-layer chromatography (TLC) profile. FRD was submitted to successive column chromatography with silica gel eluted with hexane and ethyl acetate (90:10), providing apiole (345 mg, Figure 1). The identification of apiole was accomplished by comparison of nuclear magnetic resonance (NMR 1H and 13C NMR), mass spectrometry (MS) data, and melting point with the literature [17].

### 4.2. Cell Culture

Human tumor cell lines [U251 (central nervous system, glioblastoma), MCF7 (breast, adenocarcinoma), NCI/ADR-RES (multidrug resistant ovarian adenocarcinoma), 786-0 (renal adenocarcinoma), NCI-H460 (lung, large cell carcinoma), PC-3 (prostate, adenocarcinoma), OVCAR-3 (ovarian adenocarcinoma) and HT29 (colon, adenocarcinoma)] were kindly donated by the National Cancer Institute at Frederick MA-USA. Stock cultures were grown in complete medium [RPMI 1640 medium supplemented with 5% fetal bovine serum and 1% penicillin:streptomycin mixture (1000 U∙mL^−1^:1000 µg·mL^−1^)] at 37 °C with 5% CO_2_.

### 4.3. Sample Preparation

(Doxorubicin Hydrochloride^®^—Europharma) and vincristine (Tecnocris Sulfato de Vincristina^®^—Zodiac) were prepared aseptically in DMSO (100 mg/mL) and diluted in in complete medium to afford the final concentration. The final DMSO concentration (≤0.25%) in the experiments did not affect cell viability [35].

### 4.4. Antiproliferative Activity Evaluation

Briefly, the tumor cells were seeded in 96-well plates (T1 plates, between 3 to 6 × 10^4^ cells/mL, 100 µL/well), incubated for 24 h, treated with apiole (1, 10, 100 µM and 1 mM, 100 µL/well), doxorubicin (0.046, 0.46, 4.6 and 46 µM, 100 µL/well) and vincristine (0.03, 0.3, 3 and 30 µM, 100 µL/well), in triplicate, and then incubated for 48 h at 37 °C in 5% CO_2_. A second plate, denominated T0, was prepared to infer the absorbance value of untreated cells at the moment of sample addition. Untreated (T0 and T1 plates) and treated (T1 plates) cells were fixed with 50% trichloroacetic acid and stained with sulforhodamine dye (0.4% in acetic acid 1%). Absorbance was recorded at 540 nm using a microplate reader (Molecular Devices^®^, VersaMax model). Using the absorbance values, the cell growth (%) for each cell line, at each sample concentration, was calculated considering at 100% of cell growth the difference between the absorbances of untreated cells after 48 h incubation (T1) and at the sample addition moment (T0). The curve cell growth vs. sample concentration was plotted and GI_50_ (concentration required for 50% growth inhibition) was calculated by sigmoidal regression using the Origin 8.0 software (OriginLab Corporation, Northampton, MA, USA) [36].

### 4.5. Influence of Apiole on the Antiproliferative Activity of Doxorubicin and Vincristine in Human Ovarian Tumor Lines (NCI/ADR-RES and OVCAR-3)

Following the protocol already described for the antiproliferative activity evaluation, NCI/ADR-RES and OVCAR-3 cells were seeded in 96-well plates (T1 plates, 4 and 5 × 10^4^ cells/mL, respectively, 100 µL/well). After 24 h of incubation, NCI/ADR-RES cell was treated doxorubicin (0.045, 0.45, 4.5 and 45 µM) associated with apiole at 10, 100 µM and 1 mM assay or vincristine (0.03, 0.3, 3 and 30 µM) associated with apiole at 25, 50 and 100 µM. For treatment of OVCAR-3 cells, both doxorubicin (0.045, 0.45, 4.5 and 45 µM) and vincristine (0.03, 0.3, 3 and 30 µM) were associated with apiole at 100 µM. A T0 plate was prepared for each experiment. After 48h-exposure, cell proliferation was determined as described for the antiproliferative activity evaluation.

For the analysis of the combinatorial effect, the concentration reduction index (CRI) was calculated as CRI = GI_50_^cytotoxic drug alone^/GI_50_^cytotoxic drug alone+apiol^. CRI values higher than 1 indicated synergistic combination [37].

### 4.6. Molecular Docking

The target (P-gp) for molecular docking was selected in the RCSB PDB (Protein Data Bank)—accession code 3G60, chain A, with 4.40 Å resolution. OpenBabel Cheminformatics tools of ChemInfo [38] was used to optimize the energy of the ligand (apiole), which was drawn and converted to mol.2 format, with 3D parameters and pH 7. The protein was prepared with Maestro 11.7 (Shrodinger^®^ 2018; small-molecule drug discovery suite) using Protein Preparation Wizard (Epik pH: 7.4 ± 0.5 and OPLS3e force field). The grid was defined by 20 Å × 20 Å × 20 Å box centred in the central ligand QZ59 position (co-crystal template for comparison). The ligand docking was carried out by replacing the QZ59 with Apiole, using all atoms for docking and hydrogen atoms added, considering pH 7.

### 4.7. In Silico Pharmacodynamics

The apiole structure was also submitted to ADME analysis, in order to verify the compound’s physico-chemical properties, solubility, lipophilicity, pharmacokinetics parameters (gastrointestinal and brain-blood barrier absorption and hepatic enzymes inhibition) and druglikeness, following the medicinal chemistry alert [39].

### 4.8. Statistical Analysis

Statistical analysis was performed using GraphPad Prism^®^. Comparisons among groups (three or more), considering only one independent variable/factor, were performed using One-way ANOVA followed by Tukey post-hoc tests. The results were expressed as the mean ± SD and the level of significance was 5 %.

## Figures and Tables

**Table 1:** H^1^ and C^13^ NMR spectral data for isolated compound (11 Tesla, CDCl3).

**Carbon**		^1^H	^13^C	HMBC
1	CH_2_	3.32	34.01	5, 4, 6, 7
2	CH_3_	3.84	56.78	3, 10
3	CH_3_	3.87	60.03	2, 10
4	CH_2_	5.94	101.43	5
5	CH	6.30	108.17	4
6	CH_2_	5.01	115.27	
7	C^O^	--	125.68	
8	C^O^	--	135.06	
9	C^O^	--	136.19	
10	CH	5.94	137.28	
11	C^O^	--	138.66	
12	C^O^	--	139.00	

**Table 2:** Antiproliferative activity ^a^ of apiole and the chemotherapeutic drugs doxorubicin and vincristine against human cell line panel after 48 h exposure, expressed as GI_50_^b^.

Cell Lines	Doxorubicin	Vincristine	Apiole
Central nervous system, glioblastoma (U251)	<0.045	<0.03	357.63 ± 0.25
Breast, adenocarcinoma (MCF7)	<0.045	<0.03	271.69 ± 0.53
Multidrug-resistant ovarian adenocarcinoma (NCI/ADR-RES)	4.63 ± 0.27	4.71 ± 0.27	687.63 ± 0.09
Renal adenocarcinoma (786-0)	<0.045	0.59 ± 0.10	529.87 ± 0.34
Lung, large cell carcinoma (NCI-H460)	0.04 ± 0.01	<0.03	711.04 ± 0.20
Prostate, adenocarcinoma (PC-3)	0.20 ± 0.10	0.04 ± 0.05	423.26 ± 0.14
Ovarian adenocarcinoma (OVCAR-3)	0.70 ± 0.20	1.22 ± 0.10	190.62 ± 0.24
Colon, adenocarcinoma (HT29)	0.37 ± 0.09	<0.03	n.t.

^a^ Following the NCI-60 protocol; ^b^ GI_50_ the sample concentration required (µM) to inhibit 50% of cell growth and calculated by non-linear regression analysis using ORIGIN 7.5^®^ (OriginLab Corporation); n.t. = not tested.

**Table 3:** Antiproliferative activity ^a^ expressed as GI_50_^b^ (µM) of apiole, doxorubicin, and their association against the multidrug-resistant ovarian adenocarcinoma cell line NCI/ADR-RES after 48 h exposure.

	Apiole	Doxorubicin (Doxo)	Apiole 1 mM + (Doxo)	Apiole 100 µM + (Doxo)	Apiole 10 µM + (Doxo)
GI_50_^b^	436.52 ± 27.38 ***	4.63 ± 0.61	<0.045 ***	0.55 ± 0.28 **	5.85 ± 1.60
CRI ^c^	n.a.	n.a.	>102.89	8.42	0.79

^a^ Following the NCI-60 protocol; ^b^ GI_50_ the sample concentration required (µM) to inhibit 50% of cell growth and calculated by non-linear regression analysis using ORIGIN 7.5^®^ (OriginLab Corporation); ^c^ CRI: concentration relative index calculated as GI_50_ doxorubicin/GI_50_ doxorubicin+apiole; n.a.: not applied; Concentration range evaluated: 10–1000 µM (apiole); 0.045–45 µM (doxorubicin). Statistical analysis by one-way ANOVA followed by Tukey’s test (** *p* < 0.01; *** *p* < 0.001, related to doxorubicin). The experiments were carried out in triplicate.

**Table 4:** Antiproliferative activity ^a^ expressed as GI_50_^b^ (µM) of apiole, vincristine, and their association against the multidrug-resistant ovarian adenocarcinoma cell line NCI/ADR-RES after 48 h exposure.

	Apiole	Vincristine	Apiole 100 µM + Vincristine	Apiole 50 µM + Vincristine	Apiole 25 µM + Vincristine
GI_50_^b^	>100 ***	4.71 ± 1.14	0.75 ± 0.08 **	2.70 ± 0.21 *	4.90 ± 0.77
CRI ^c^	n.a.	n.a.	6.28	1.74	0.96

^a^ Following the NCI-60 protocol; ^b^ GI_50_: the sample concentration required (µM) to inhibit 50% of cell growth and calculated by non-linear regression analysis using ORIGIN 7.5^®^ (OriginLab Corporation); ^c^ CRI: concentration relative index calculated as GI_50_ vincristine/GI_50_ vincristine+apiole; n.a.: not applied; Concentration range evaluated: 25–100 µM (apiole); 0.03–30 µM (vincristine). Statistical analysis by one-way ANOVA followed by Tukey’s test (* *p* < 0.05; ** *p* < 0.01; *** *p* < 0.001, related to vincristine). The experiments were carried out in triplicate.

**Table 5:** Antiproliferative activity ^a^ expressed as GI_50_^b^ (µM) of doxorubicin, vincristine, and their associations with apiole against the ovarian adenocarcinoma cell line OVCAR-3 after 48 h exposure.

	Doxorubicin	Apiole 100 µM + Doxorubicin	Vincristine	Apiole 100 µM + Vincristine
GI_50_^b^	0.70 ± 0.10	0.32 ± 0.11 **	1.22 ± 0.19	0.43 ± 0.12 **
CRI ^c^	n.a.	2.2	n.a.	2.8

^a^ Following the NCI-60 protocol; ^b^ GI_50_: the sample concentration required (µM) to inhibit 50% of cell growth and calculated by non-linear regression analysis using ORIGIN 7.5^®^ (OriginLab Corporation); ^c^ CRI: concentration relative index calculated as GI_50_ drug/GI_50_ drug+apiole; n.a.: not applied; Concentration range evaluated: 1.25 to 20 μM for doxorubicin and vincristine. Statistical analysis by one-way ANOVA followed by Tukey’s test (* *p* < 0.05; ** *p* < 0.01; *** *p* < 0.001, related to vincristine). The experiments were carried out in triplicate.

**Table 6:** Pharmacokinetics parameters predicted for apiole in comparison to Lipinski’s criteria.

Parameter	Criteria	Apiole
Molecular weight	<500 g/mol	222.24 g/mol
Hydrogen-bond acceptors (HBA)	<10	4
Hydrogen-bond donors (HBD)	<5	0
LogP	2 a 5	2.85
Topological polar surface area (TPSA)	40 a 100 A^2^	36.92 A^2^
Gastrointestinal absorption	Yes	Yes (high)
Blood-brain barrier permeant	No	Yes
Metabolism enzymes inhibitors	<2	1

## References

[1] Bray F., Ferlay J., Soerjomataram I., Siegel R.L., Torre L.A., Jemal A. (2018). Global cancer statistics 2018: GLOBOCAN estimates of incidence and mortality worldwide for 36 cancers in 185 countries. CA Cancer J. Clin..

[2] Longley D.B., Johnston P.G. (2005). Molecular mechanisms of drug resistance. J. Pathol..

[3] Vert A., Castro J., Ribó M., Vilanova M., Benito A. (2018). Transcriptional profiling of NCI/ADR-RES cells unveils a complex network of signaling pathways and molecular mechanisms of drug resistance. Onco Targets Ther..

[4] Li W., Zhang H., Assaraf Y.G., Zhao K., Xu X., Xie J., Yang D.H., Chen Z.S. (2016). Overcoming ABC transporter-mediated multidrug resistance: Molecular mechanisms and novel therapeutic drug strategies. Drug Resist. Updat..

[5] Dong J., Qin Z., Zhang W.D., Cheng G., Yehuda A.G., Ashby C.R., Chen Z.S., Cheng X.D., Qin J.J. (2020). Medicinal chemistry strategies to discover P-glycoprotein inhibitors: An update. Drug Resist. Updat..

[6] Robey R.W., Pluchino K.M., Hall M.D., Fojo A.T., Bates S.E., Gottesman M.M. (2018). Revisiting the role of ABC transporters in multidrug-resistant cancer. Nat. Rev. Cancer.

[7] Sodani K., Patel A., Kathawala R.J., Chen Z.S. (2012). Multidrug resistance associated proteins in multindrung resistance. Chin. J. Cancer.

[8] Callaghan R., Luk F., Bebawy M. (2014). Special Section on Transporters in Toxicity and Disease—Commentary The Role of Transporters in Toxicity and Disease. Drug Metab. Dispos..

[9] da Silva E.M.J. (1997). Morfologia e ontogênese das estruturas secretoras em Piper regnellii (Miq.) C. DC. var. regnellii: Piperaceae. Acta Bot. Brasilica.

[10] Prinsloo G., Nogemane N., Street R. (2018). The use of plants containing genotoxic carcinogens as foods and medicine. Food Chem. Toxicol..

[11] Lai Y.Y., Lien H.M., Kuo P.T., Huang C.L., Kao J.Y., Lin H., Yang D.Y. (2011). Study of the anti-proliferative activity of 5-substituted 4,7-dimethoxy-1,3-benzodioxole derivatives of sy-1 from Antrodia camphorata on human COLO 205 colon cancer cells. Evidence -Based Complement. Altern. Med..

[12] Wei P.L., Tu S.H., Lien H.M., Chen L.C., Chen C.S., Wu C.H., Huang C.-S., Chang H.-W., Chang C.-H., Ho Y.-S. (2012). The in vivo antitumor effects on human COLO 205 cancer cells of the 4,7-dimethoxy-5-(2-propen-1-yl)-1,3-benzodioxole (apiole) derivative of 5-substituted 4,7-dimethoxy-5-methyl-l,3-benzodioxole (SY-1) isolated from the fruiting body of Antrodia camphorate. J. Cancer Res. Ther..

[13] Wu K.H., Lee W.J., Cheng T.C., Chang H.W., Chen L.C., Chen C.C., Lien H.-M., Lin T.-N., Ho Y.-S. (2019). Study of the antitumor mechanisms of apiole derivatives (AP-02) from Petroselinum crispum through induction of G0/G1 phase cell cycle arrest in human COLO 205 cancer cells. BMC Complement. Altern. Med..

[14] Neuhaus-Carlisle K., Vierling W., Wagner H. (1993). Calcium-channel blocking activity of essential oils from Petroselinum crisp., Api graveolens and isolated phenylpropane constituents. Pharm. Pharmacol. Lett..

[15] Neuhaus-Carlisle K., Vierling W., Wagner H. (1997). Screening of plant extracts and plant constituents for calcium-channel blocking activity. Phytomedicine.

[16] Micucci M., Viale M., Chiarini A., Spinelli D., Frosini M., Tavani C., Maccagno M., Bianchi L., Gangemi R., Budriesi R. (2020). 3-Aryl-4-nitrobenzothiochromans S,S-dioxide: From calcium-channel modulators properties to multidrug-resistance reverting activity. Molecules.

[17] Benevides P.J.C., Sartorelli P., Kato M.J. (1999). Phenylpropanoids and neolignans from Piper regnellii. Phytochemistry.

[18] Kim S., Chen J., Cheng T., Gindulyte A., He J., He S., Li Q., Shoemaker B.A., Thiessen P.A., Yu B. (2019). PubChem 2019 update: Improved access to chemical data. Nucleic Acids Res..

[19] Palmeira A., Sousa E., Vasconcelos M.H., Pinto M.M. (2012). Three Decades of P-gp Inhibitors: Skimming Through Several Generations and Scaffolds. Curr. Med. Chem..

[20] Jagodinsky J.C., Akgun U. (2015). Characterizing the binding interactions between P-glycoprotein and eight known cardiovascular transport substrates. Pharmacol. Res. Perspect..

[21] David M.A., Orlowski S., Prichard R.K., Hashem S., André F., Lespine A. (2016). In silico analysis of the binding of anthelmintics to Caenorhabditis elegans P-glycoprotein 1. Int. J. Parasitol. Drugs Drug Resist..

[22] Lipinski C.A., Lombardo F., Dominy B.W., Feeney P.J. (2012). Experimental and computational approaches to estimate solubility and permeability in drug discovery and development settings. Adv. Drug Deliv. Rev..

[23] Lepeltier E., Rijo P., Rizzolio F., Popovtzer R., Petrikaite V., Assaraf Y.G., Passirani C. (2020). Nanomedicine to target multidrug resistant tumors. Drug Resist. Updat..

[24] Huber P.C., Maruiama C.H., Almeida W.P. (2010). Glicoproteína-P, resistência a múltiplas drogas (MDR) e relação estrutura-atividade de moduladores. Quim. Nova.

[25] Adovelande J., Delèze J., Schrével J. (1998). Synergy between two calcium channel blockers, verapamil and fantofarone (SR33557), in reversing chloroquine resistance in Plasmodium falciparum. Biochem. Pharmacol..

[26] Oneschuk D., Younus J. (2008). Natural health products and cancer chemotherapy and radiation therapy. Oncol. Rev..

[27] Liscovitch M., Ravid D. (2007). A case study in misidentification of cancer cell lines: MCF-7/AdrR cells (re-designated NCI/ADR-RES) are derived from OVCAR-8 human ovarian carcinoma cells. Cancer Lett..

[28] Hansch C., Selassie C. (2007). Quantitative Structure-activity relationship—A historical perspective and the future. Compr. Med. Chem. II.

[29] Menichetti R., Kanekal K.H., Bereau T. (2019). Drug-Membrane Permeability across Chemical Space. ACS Cent. Sci..

[30] Zhou G.-D., Moorthy B., Bi J., Donnelly K.C., Randerath K. (2007). DNA Adducts From Alkoxyallylbenzene Herb and Spice Constituents in Cultured Human (HepG2) Cells. Environ. Mol. Mutagen..

[31] Samet A.V., Shevchenko O.G., Rusak V.V., Chartov E.M., Myshlyavtsev A.B., Rusanov D.A., Semenova M.N., Semenov V.V. (2019). Antioxidant Activity of Natural Allylpolyalkoxybenzene Plant Essential Oil Constituents. J. Nat. Prod..

[32] Callaghan R., Luk F., Bebawy M. (2014). Inhibition of the multidrug resistance P-glycoprotein: Time for a change of strategy?. Drug Metab. Dispos..

[33] Liu Y., Lu Z., Fan P., Duan Q., Li Y., Tong S., Hu B., Lv R., Hu L., Zhuang J. (2011). Clinical Efficacy of Chemotherapy Combined with Verapamil in Metastatic Colorectal Patients. Cell Biochem. Biophys.

[34] Gadhe C.G., Cho S.J. (2011). Modulation of Multidrug Resistance in Cancer by P-Glycoprotein Modulation of Multidrug Resistance in Cancer by P-Glycoprotein. J. Chosun. Nat. Sci..

[35] Banzato T.P., Gubiani J.R., Bernardi D.I., Nogueira C.R., Monteiro A.F., Juliano F.F., De Alencar S.M., Pilli R.A., De Lima C.A.D., Longato G.B. (2020). Antiproliferative Flavanoid Dimers Isolated from Brazilian Red Propolis. J. Nat. Prod..

[36] Franco Y.E.M., Okubo M.Y., Torre A.D., Paiva P.P., Rosa M.N., Silva V.A.O., Reis R.M., Ruiz A.L.T.G., Imamura P.M., de Carvalho J.E. (2019). Coronarin D induces apoptotic cell death and cell cycle arrest in human glioblastoma cell line. Molecules.

[37] Chou T.C. (2010). Drug combination studies and their synergy quantification using the chou-talalay method. Cancer Res..

[38] ChemInfo. http://www.cheminfo.org/.

[39] Daina A., Michielin O., Zoete V. (2017). SwissADME: A free web tool to evaluate pharmacokinetics, drug-likeness and medicinal chemistry friendliness of small molecules. Sci. Rep..

